# Outcome of childhood bacterial meningitis on three continents

**DOI:** 10.1038/s41598-021-01085-w

**Published:** 2021-11-03

**Authors:** Heikki Peltola, Irmeli Roine, Markku Kallio, Tuula Pelkonen

**Affiliations:** 1 Pediatrics, University of Helsinki and Helsinki University Hospital, P.O. Box 22, 00014 University of Helsinki, Finland; 2 Faculty of Medicine, University Diego Portales, Santiago, Chile; 3 New Children’s Hospital, Pediatric Research Center, P.O. Box 347, 00029 HUS Helsinki, Finland; 4 Hospital Pediátrico David Bernardino, Luanda, Angola

**Keywords:** Bacterial infection, Meningitis, Microbiology, Diseases, Medical research, Neurology

## Abstract

Our objective was to quantify the differences in the outcomes from childhood bacterial meningitis (BM) and to describe the factors associated with them in different parts of the world. This study is a secondary analysis of prospectively collected data from five clinical BM trials conducted in Finland, Latin America (LatAm), and Angola between 1984 and 2017. As all data were collected uniformly, direct comparison of the series was possible. Associations between patient characteristics and death or dismal outcome—the triad of death, severe neurological sequelae, or deafness—were explored. In all, data on 2123 children with BM were analyzed. Etiology was confirmed in 95%, 83%, and 64%, in Finland, LatAm and Angola, respectively. The leading agents were *Haemophilus influenzae*, *Streptococcus pneumoniae,* and *Neisseria meningitidis*. Dismal outcome was the end result for 54%, 31%, and 5% of children in Angola, LatAm, and Finland, respectively. Although underweight, anemia, and tardy arrival worsened prognoses in Angola and LatAm, it was the presenting condition that was central in terms of outcome. In multivariate analysis, the factors independently associated with dismal outcome were the study site (Angola *vs.* Finland, OR 11.91, 95% CI 5.54–25.63, p < 0.0001 or LatAm *vs.* Finland, OR 9.46, 95% CI 4.35–20.61, p < 0.0001), Glasgow Coma Score < 13 (OR 4.58, 95% CI 3.31–6.32, p < 0.0001), seizures (OR 1.96, 95% CI 1.43–2.69), age < 1 year (OR 1.55, 95% CI 1.13–2.14, p = 0.007), and pneumococcal etiology (OR 1.49, 95% CI 1.08–2.06, p = 0.015). Greatly dissimilar outcomes from BM reflected the findings on admission on all three continents. Optimizing growth, preventing anemia, and prompt treatment may improve outcomes in resource poor areas.

## Introduction

Despite effective *Haemophilus influenzae* type b (Hib), *Streptococcus pneumoniae,* and *Neisseria meningitidis* vaccinations in many countries, bacterial meningitis (BM) remains a major problem in children across the world^1^. It is the 10th most common cause of death among those under 5 years old, with around 150.000 succumbing from BM annually^2^. Unfavorable outcomes are especially common in Africa^3^ and Latin America (LatAm)^4^. The topicality of BM is by no means over.

With the aim of easing the burden of childhood BM using treatments sufficiently straightforward to deploy in resource-poor settings, our group has carried out five large, prospective randomized trials on three continents. The then new third-generation cephalosporins were first compared with chloramphenicol and ampicillin in Finland in 1984–1991^5^. No major difference in performance was found.

In the second trial ceftriaxone was used, mainly because it is simple to administer. This study focused on the adjuvant dexamethasone *versus* oral glycerol^6^, an agent used to reduce increased intracranial pressure especially after neurosurgery^7,8^. Glycerol showed encouraging effects, but the study had to be curtailed as the Hib polysaccharide^9^ and conjugate^10^ vaccines arrived to eliminate the leading agent of pediatric BM in Finland^11^. Nevertheless, those two studies included 351 cases.

The third trial was carried out in six countries of LatAm (Argentina, Brazil, Dominican Republic, Ecuador, Paraguay, and Venezuela) in 1995–2003^12–14^. The main lesson from the largest (N 654) ever randomized BM study performed at the time was that severe neurological sequelae were prevented with glycerol (in recipients 15/276 *vs.* non-recipients 29/273, odds ratio 0.5, p 0.02), but not with dexamethasone alone^12^. This important observation obliged us ethically to use glycerol as adjuvant in our subsequent trials.

Finally, two studies with a total of 1118 patients took place in Luanda, Angola, in 2005–2017^15–17^. In the first study cefotaxime was given by bolus or slow infusion, with participants also receiving concomitantly, at random, oral acetaminophen (paracetamol), or placebo. Our interest in paracetamol was raised by positive effects obtained in bacteremic adult patients^18^. The series of 723 children^15^ showed highly significant benefit with the regimen of oral paracetamol plus cefotaxime infusion during six to 600 h from institution of therapy, but significance was lost afterwards. The second Angolan study on 375 patients^17^, in which a prolonged cefotaxime infusion plus paracetamol regimen was tested, failed to confirm these encouraging results.

These five prospective BM studies generated an exceptionally large dataset. Notwithstanding the prolonged timespan, having the same information on the same devastating disease in dissimilar conditions on three continents offered the possibility that overall analysis of this data would allow us to observe conclusive trends. Our hypothesis was that besides the children's general condition upon arrival at hospital, local conditions would play a major role in their prognosis.

## Methods

### Trial designs, participants, and diagnosis

The clinical trial data used in this manuscript has been published before^5,6,12,15,17^. The setup of each study has been detailed earlier^5,6,12,15,17^. In short, after approval of the protocol by the relevant Ethics Committees, children aged from two months to 15 years with symptoms and signs compatible with BM were included, after obtaining consent from the legal guardian. In case of illiteracy, a finger print was required. The study outline was explained to guardians by the attending physician. Using a computer-generated list, participants were randomized to different groups according to the protocol. All methods were carried out in accordance with the Declaration of Helsinki. After becoming a practice, the two Angolan studies were registered with the International Standard Randomized Controlled Trial Number Register, number ISRCTN62824827, 22 August 2005, and with ClinicalTrials.gov, identifier NCT 01540838, 29 February 2012, respectively. In Finland, the patients were enrolled between 30 October 1984 and 19 December 1986, and between 21 April 1987 and 11 November 1990, in LatAm between 10 January 1996 and 20 December 2003, and in Luanda between 18 July 2005 and 26 June 2008, and between 22 January 2012 and 21 January 2017.

While examining the patient, the on-call doctor assessed the child's condition using the age-adjusted Glasgow Coma Scale, performed a lumbar puncture, ordered pre-determined samples, instituted treatment according to the protocol, and began to complete the specially designed forms. The questionnaires were in Finnish, Spanish, or Portuguese, depending on the location. Basic bacteriology and chemistry were performed in local laboratories using standard techniques.

BM was deemed confirmed if (1) cerebrospinal fluid (CSF) culture proved positive, (2) patient showed compatible symptoms and signs and a positive blood culture, or (3) at least two of the following criteria were fulfilled: CSF leukocytes ≥ 100/mm^3^ (predominantly polymorphs), positive Gram stain, positive latex agglutination test, or serum C-reactive protein (CRP) ≥ 40 mg/Liter. Exclusion criteria were age less than two months, trauma, intracranial shunt, previous hearing impairment or neurological disease, and immunosuppression, except in potential HIV-infection (relevant mainly in Angola). Pretreatment antimicrobials prevented enrollment if more than one parenteral dose had been administered. Malnourishment was graded according to z-scores following WHO guidelines^14^.

### Monitoring the disease, analysis of data

Data were compiled on the ward administering the treatment. The course of illness was monitored daily using the dedicated forms. Since consecutive CRP measurements offer a yardstick for monitoring BM^19^, CRP was quantified by at least day 4 (+/− 1 day), after which values exceeding 62 mg/l are associated with slow recovery and problems such as hearing impairment^20^. After the child was discharged from hospital, all data were sent to Finland to be digitized and analyzed.

Outcomes were assessed at discharge. Between uneventful recovery and death as the extreme outcomes there were further categories: survival with mild hearing deficit (better ear threshold 41–79 dB), deafness (threshold ≥ 80 dB), and mild or severe neurological sequelae. Sequelae were deemed mild if the patient showed any abnormality, such as hemiparesis, monoparesis, psychomotor retardation, or ataxia, whereas severe sequelae included blindness, quadriplegia/paresis, hydrocephalus requiring a shunt, or severe psychomotor retardation. Finally, the triad of severe neurological sequelae, deafness, or death comprised a dismal outcome. All these categories are summarized in the footnote of Table 1. The best available audiological techniques were used, with traditional audiometry recommended. For small children, brain evoked response audiometry (BERA) was the preferred method of choice.

**Table 1:** Patient characteristics at arrival and during illness, and outcomes.

	Whole series N = 2123	Finland N = 351	Latin America N = 654	Angola N = 1118	*p* Value
**At arrival**					
Females	942/2123 (44)^a^	158/351 (45)	281/654 (43)	503/1118 (45)	0.69
Age—yr, median	1.3 (0.6–3.9)	1.8 (0.8–3.8)	0.9 (0.5–3.6)	1.4 (0.6–4.2)	< 0.0001
Axillary temperature—°C	38.0 (37.0–38.8)	39.0 (38.3–39.7)	38.0 (37.0–38.6)	37.6 (37.6–38.3)	< 0.0001
Ill before arrival—days	4.0 (2.0–7.0)	1.2 (0.8–2.0)	3.0 (2.1–5.0)	5.0 (3.0–7.0)	< 0.0001
Pretreatment antibiotics	723/1960 (37)	62/340 (18)	214/589 (36)	447/1031 (43)	< 0.0001
Seizures before/at arrival	842/2057 (41)	62/345 (18)	213/610 (35)	567/1102 (51)	< 0.0001
Altered consciousness	1504/2065 (73)	266/351 (76)	458/609 (75)	780/1105 (71)	0.048
Glasgow Coma Score < 13	1035/2063 (50)	138/346 (40)	249/609 (41)	648/1108 (58)	< 0.0001
Hb < 8.5 g/dL	966/1896 (51)	4/341 (1)	208/598 (35)	728/1085 (67)	< 0.0001
Weight/age z-score < -2	435/2053 (21)	10/313 (3)	78/624 (13)	347/1116 (31)	< 0.0001
CSF leukocyte count/mm^3^	1350 (262–4000)	3100 (1270–7200)	1950 (632–6300)	750 (150–2500)	< 0.0001
CSF glucose, mg/dL	17.6 (7.3–40.6)	34.2 (12.6–61.3)	15.0 (5.0–40.0)	15.5 (7.5–33.0)	< 0.0001
CSF protein, mg/dL	170 (99–256)	166 (96–257)	158 (89–255)	193 (113–261)	0.059
**During illness course, outcomes**					
Bacteria identified	1597/2123 (75)	334/351 (95)	543/654 (83)	720/1118 (64)	< 0.0001
Etiology of bacterial meningitis					< 0.0001
*Streptococcus pneumoniae*	506 (31.7)	28/334 (8.4)	144/543 (26.5)	334/720 (46.4)	
*Haemophilus influenzae*	668 (41.8)	218/334 (65.2)	243/543 (44.8)	207/720 (28.7)	
*Neisseria meningitidis*	285 (17.9)	80/334 (24.0)	115/543 (21.2)	90/720 (12.5)	
Other bacteria	138 (8.6)	8/334 (8.4)	41/543 (7.5)	89/720 (12.4)	
CRP, day 4 (+ /- 1)—mg/L	60 (30–106)	46 (31–81)	47 (18–89)	90 (40–150)	< 0.0001
Days in hospital	10 (8–12)	11 (9–11)	8 (7–11)	11 (9–16)	< 0.0001
Mild hearing deficit^b^	250/1027 (24)	21/283 (7)	103/405 (25)	126/339 (37)	< 0.0001
Deafness^c^	113/1368 (8)	0/283 (0)	49/454 (11)	64/631 (10)	< 0.0001
Mild neur. sequelae^d^	355/1417 (25)	54/335 (16)	104/502 (21)	197/580 (34)	< 0.0001
Severe neur. sequelae^e^	147/1575 (9)	4/339 (1)	43/547 (8)	100/689 (15)	< 0.0001
Mild sequelae^f^	364/936 (39)	54/280 (19)	133/366 (36)	177/290 (61)	< 0.0001
Severe sequelae^g^	231/1372 (17)	4/264 (1)	81/448 (18)	146/640 (23)	< 0.0001
Dismal outcome^h^	755/1896 (40)	16/296 (5)	168/535 (31)	571/1065 (54)	< 0.0001
Death	524/2123 (25)	12/351 (3)	87/654 (13)	425/1118 (38)	< 0.0001

^a^Data is presented in n/N (%) or median (interquartile range, 25–75 percentile), whichever relevant.

^b^Better ear's hearing threshold 41–79 dB.

^c^Better ear's hearing threshold ≥ 80 dB.

^d^Other than severe neurological sequelae.

^e^Blindness, quadriplegia/paresis, hydrocephalus requiring a shunt, or severe psychomotor retardation.

^f^Other than severe sequelae.

^g^Deafness or severe neurological sequelae.

^h^Death or severe neurological sequelae or deafness.

All data were computed and analyzed using JMP ® Pro 14.1.0 (SAS Institute Inc, Cary, NC, USA) for Windows. Contingency analysis was used to examine relationships between two categorial variables, and Pearson´s chi-square test to calculate p values. Associations with continuous characteristics were assessed using One-Way Anova. We used nominal logistic analysis and calculated odds ratios (OR) with 95% confidence intervals (95% CI) for death and dismal outcome. For multivariate analysis of prognostic factors, we used clinical characteristics that in univariate analysis showed p value < 0.0001.

### Ethics approval

All the studies were approved by the relevant Ethics Committees or the Hospitals’ Board. The children were enrolled after their guardian´s informed consent was obtained.

## Results

### Patient characteristics on arrival

The total number of cases was 2123, of which 1597 (75%) were confirmed bacteriologically. The Finnish series comprised 351 (confirmed 334, 95%) cases, the LatAm series 654 (543, 83%) cases, and the two trials in Angola 1118 (720, 64%) cases. Genders presented fairly evenly overall: 56% were boys and 44% girls. Also, the age distribution of the three series was rather similar, except that while the majority in LatAm and Angola were infants, the one-year-olds slightly preponderated in Finland (*p* < 0.0001).

Table 1 characterizes the patients by 21 covariates and outcomes. There were significant differences between the sites, except in gender distribution (*p* = 0.69). Altered consciousness, an important signal of degree of inflammation in the central nervous system, was recorded in 71–76% across the series (*p* = 0.048). Regarding the Glasgow Coma Scale scores, levels under 13, which indicates severe disease, were shown by 40–41% of patients in Finland and LatAm *vs.* by 58% of those in Angola (*p* < 0.0001).

Some characteristics in Table 1 deserve further attention. Median time with symptoms and signs suggestive of commencing or overt BM was 4.0 (quartile 2.0–7.0) days overall, though this varied significantly from 1.2 (0.8–2.0) days in Finland, to 3.0 (2.1–5.0) days in LatAm, and 5.0 (3.0–7.0) days in Angola (*p* < 0.0001). Pretreatment antibiotics had been given to 18% of children in Finland compared to 36% in LatAm and 43% in Angola (*p* < 0.0001). The agents used were mostly oral β-lactams, such as amoxicillin (data not shown).

Anemia was common in Angola and LatAm. A median hemoglobin level of 8.5 g/dL was not achieved by 67% of Angolan and 35% of LatAm patients, compared to just one percent in Finland (*p* < 0.0001). A similar trend was observed in the weight for age z-score of below -2, which suggests at least moderate underweight. This was the case for 31% of children in Angola, 13% in LatAm and 3% in Finland (*p* < 0.0001).

In Angola, 57 (8%) of 737 children had positive HIV antibody test result. Malaria thick film was positive in 29% (311/1055) of Angolan children. In Latin America and Finland there were no known cases with HIV or malaria.

### Findings during treatment, outcomes

Etiology reflected the continent (Table 1). Initially, Hib was the leading causative agent in Finland and LatAm, with 218 (65%) of 334 bacteriologically identified cases *vs.* 243 (45%) of 543 cases, respectively. In Angola, *H. influenzae* was not invariably typed, but once done, Hib predominated overwhelmingly. There *S. pneumoniae* dominated with 334 (46%) of the 720 confirmed cases. *N. meningitidis* ranked second in Finland and third in LatAm and Angola, the respective case numbers being 80, 115, and 90.

**Figure 1:**
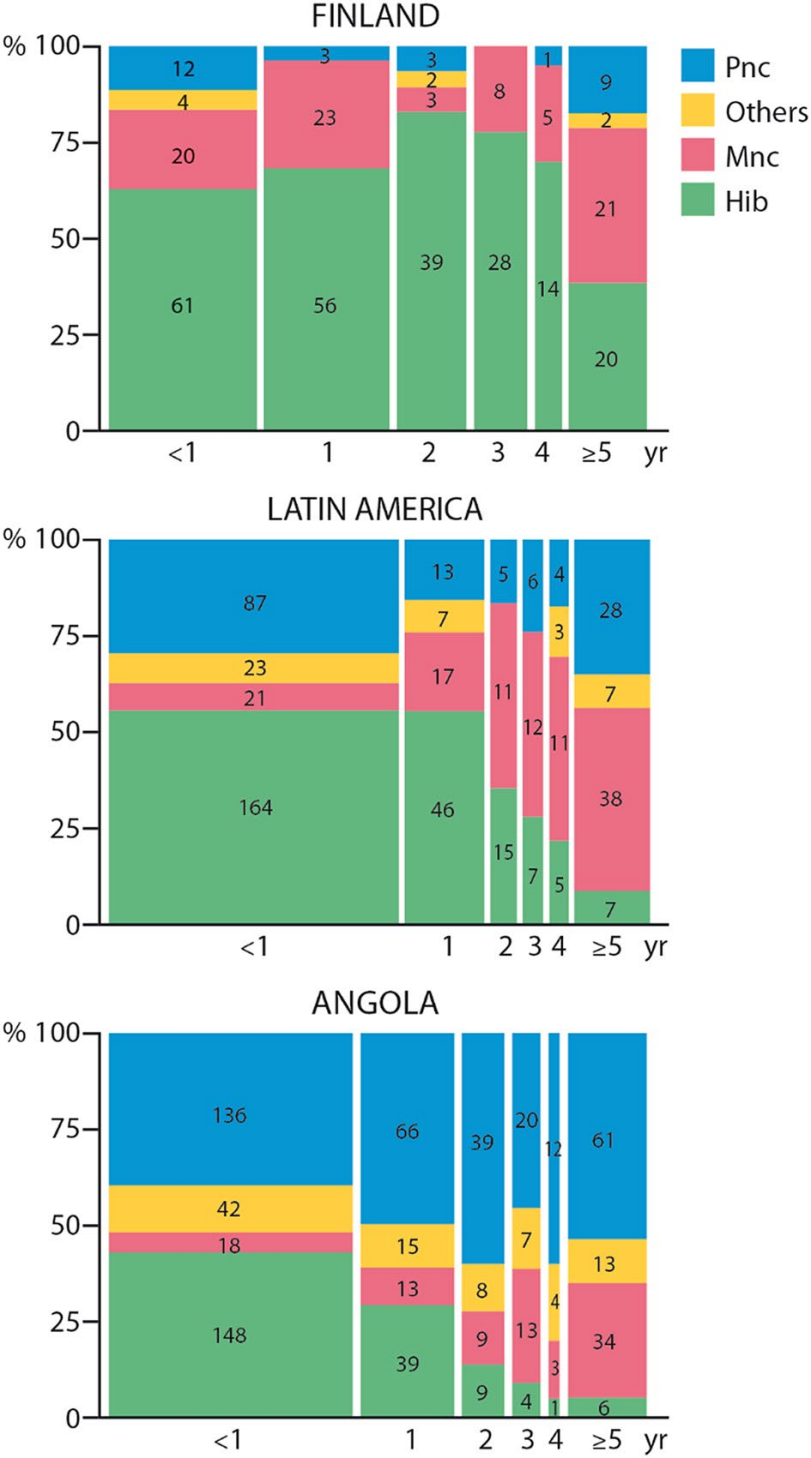
The etiology as a function of age in Finland, Latin America, and Angola. *Pnc* *Streptococcus pneumoniae;* Others denotes mainly Gram-negative rods; *Mnc* *Neisseria meningitides*, *Hib* *Haemophilus influenzae* type b.

A change in etiology occurred over time. The year of BM diagnosis was earlier for patients with *H. influenzae* or *N. meningitidis* than for patients with *S. pneumoniae* or other bacteria (*p* < 0.0001). This was the case also when all the areas were analyzed separately (*p* < 0.001). Figure 1 shows etiology in the three series as a function of age.

The highest CRP values around day four were observed in Angola (*p* < 0.0001) (Table 1). This was an omen of the poorer outcomes there^20^. Length of hospital stay did not differ greatly, the median being 11 days in Angola and Finland *vs.* 8 days in LatAm (*p* < 0.0001).

**Figure 2:**
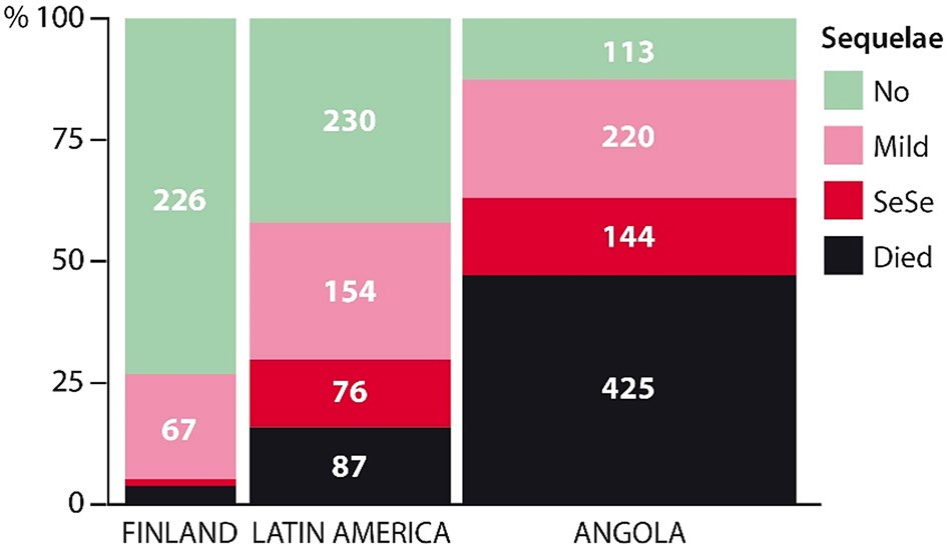
Main outcomes of 309, 547 and 902 cases from Finland, Latin America and Angola, respectively. Mild and severe sequelae (SeSe) defined in text. Bacteriologically confirmed and unconfirmed cases combined.

Overall, 524 (25%) children died, one-quarter of the entire series, the percentages being 3, 13, and 38 in Finland, LatAm, and Angola, respectively (*p* < 0.0001). Of the total 1599 survivors, 1234 (77%) were fully neurologically and audiologically examined, of whom only 569 (46%) recovered uneventfully, whereas 441 (36%) survived with mild, and 224 (18%) with severe sequelae. In Finland the respective figures were 226/297 (76%), 67 (23%) and 4 (1%); in LatAm 230/460 (50%), 154 (33%), and 76 (17%)*;* and in Angola 113/477 (24%), 220 (46%), and 144 (30%) (*p* < 0.0001). Figure 2 summarizes the overall outcomes at the three investigation sites. There were major differences between North and South in terms of uneventful recovery and death.

Total deafness, a daunting disorder especially for a child in the developing world, hit 10–11% of those in Angola and LatAm, compared to none in Finland (*p* < 0.0001). Similarly, 15–8% of patients in Angola and LatAm were left with severe neurological sequelae *vs.* one percent in Finland (*p* < 0.0001). Dismal outcome (death, severe neurological sequelae, or deafness) was the fate of 54%, 31%, and 5% of patients in Angola, LatAm, and Finland, respectively (*p* < 0.0001) (Table 1).

**Figure 3:**
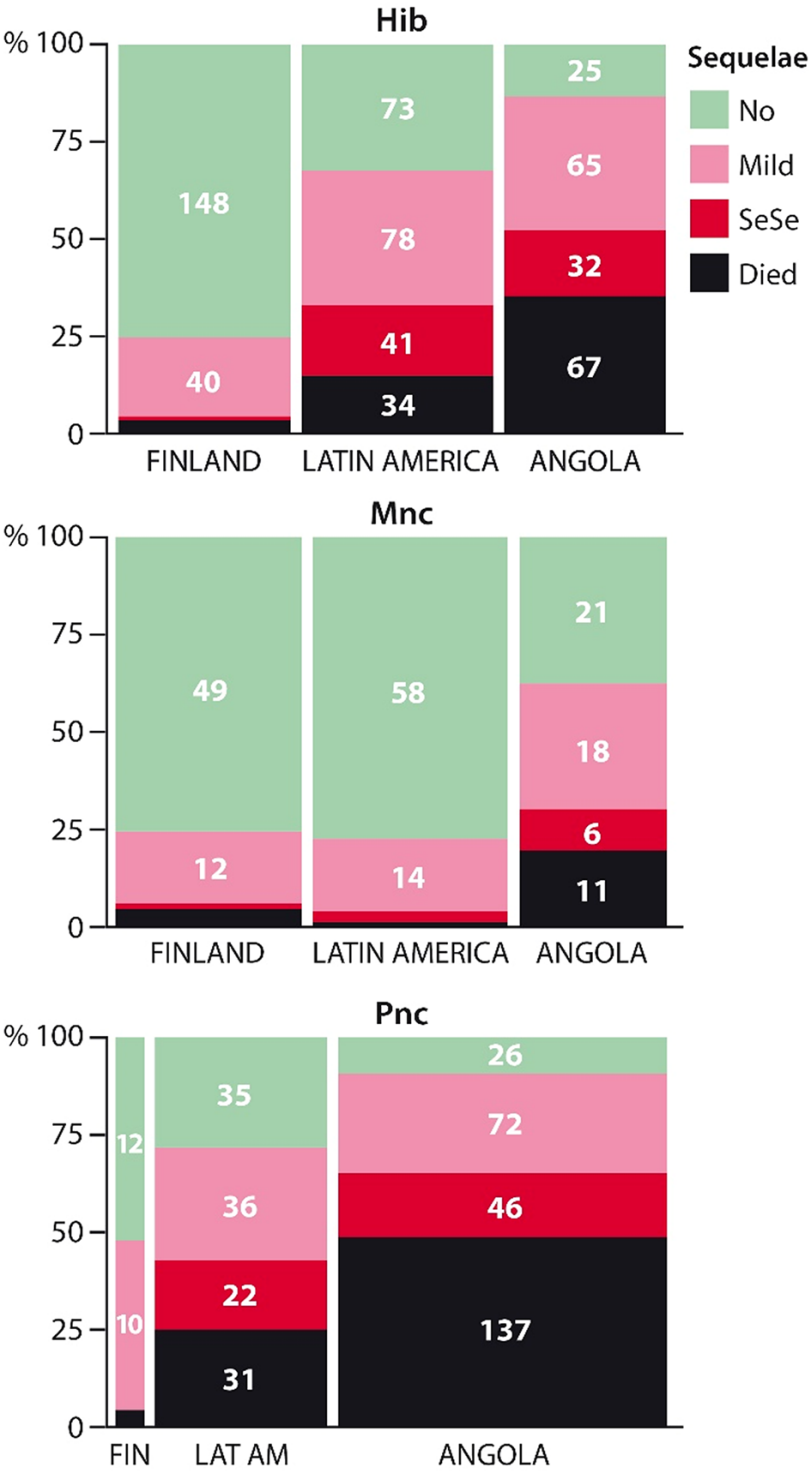
Outcomes of childhood bacterial meningitis according to the main etiology and the study site. *Pnc* *Streptococcus pneumoniae*, Others denotes a group comprising mainly Gram-negative rods, *Mnc* *Neisseria meningitidis*, *Hib* *Haemophilus influenzae* type b. Mild and severe sequelae (SeSe) explained in text.

Figure 3 shows the outcomes of 1435 children site-wise. Pneumococcal meningitis was especially deleterious in Angola, with a fatality rate of 41% (137/334), *vs.* 22% (31/144) in LatAm and 4% (1/27) in Finland (*p* < 0.0001). The overall mortality from pneumococcal meningitis was 33% (169/505).

### Associations with death and dismal outcome

Children scoring low on the Glasgow Coma Scale, and those seizing at arrival or with a seizure history showed the highest risk of succumbing (Table 2). For scores under 13, the odds ratios were 4.21, 6.57, and 4.23, in Finland, LatAm and Angola, respectively. Similarly, for children having seizure prior to or on admission, the odds ratios were 8.88, 3.50, and 2.37, respectively.

**Table 2:** Odds ratios with 95% confidence intervals of variables related to death or dismal outcome, univariate analysis.

	Finland	LatAm	Angola	Whole series
	OR (95% CI)	OR (95% CI)	OR (95% CI)	OR (95% CI)
**Death**	0.03 (0.02–0.06)	0.13 (0.11–0.16)	0.38 (0.35–0.41)	0.25 (0.23–0.27)
Age < 1 year	1.26 (0.37–4.30)	1.93 (1.21–3.13)	0.77 (0.60–0.99)	0.96 (0.78–1.17)
Ill before arrival > 3 days	0.58 (0.07–4.70)	2.47 (1.28–4.76)	2.06 (1.58–2.70)	3.30 (2.63–4.15)
Pretreatment antibiotics	0.40 (0.05–3.14)	1.21 (0.74–1.98)	1.42 (1.10–1.83)	1.69 (1.37–2.08)
Weight/age z-score < -2	5.50 (0.60–50.56)	2.60 (1.47–4.60)	1.48 (1.15–1.92)	2.54 (2.03–3.18)
Seizures before/at arrival	8.88 (2.51–31.36)	3.50 (2.16–5.66)	2.37 (1.85–3.04)	3.55 (2.88–4.38)
Altered consciousness	3.62 (0.46–28.48)	5.90 (2.34–14.87)	5.01 (3.60–6.99)	3.97 (2.95–5.35)
Glasgow Coma Score < 13	4.21 (1.10–16.14)	6.57 (3.78–11.42)	4.23 (3.21–5.58)	5.22 (4.13–6.58)
Blood hemoglobin < 8.5 g/dl	-^**a**^	2.14 (1.32–3.46)	1.06 (0.82–1.38)	2.52 (2.04–3.11)
*S. pneumoniae* etiology	1.13 (0.14–9.15)	2.02 (1.22–3.34)	1.50 (1.10–2.04)	2.54 (1.99–3.24)
**Dismal outcome**^b^	0.05 (0.03–0.09)	0.31 (0.28–0.35)	0.54 (0.51–0.57)	0.40 (0.38–0.42)
Age < 1 year	2.13 (0.76–5.91)	2.71 (1.83–4.02)	1.39 (1.08–1.78)	1.60 (1.33–1.93)
Ill before arrival > 3 days	1.49 (0.39–5.65)	2.35 (1.42–3.89)	2.19 (1.69–2.83)	3.37 (2.73–4.16)
Pretreatment antibiotics	0.30 (0.04–2.34)	1.23 (0.83–1.82)	1.84 (1.42–2.38)	1.91 (1.57–2.32)
Weight/age z-score < -2	7.81 (1.42–43.03)	1.89 (1.13–3.16)	1.55 (1.19–2.01)	2.44 (1.95–3.05)
Seizures before/at arrival	6.87 (2.28–20.77)	3.33 (2.25–4.92)	2.76 (2.15–3.54)	3.79 (3.12–4.61)
Altered consciousness	2.33 (0.52–10.52)	3.61 (2.05–6.37)	5.43 (4.04–7.32)	3.72 (2.91–4.77)
Glasgow Coma Score < 13	1.68 (0.59–4.77)	4.20 (2.82–6.25)	5.32 (4.07–6.94)	4.89 (3.99–6.01)
Blood hemoglobin < 8.5 g/dl	–	2.60 (1.76–3.85)	1.28 (0.99–1.66)	2.96 (2.43–3.59)
*S. pneumoniae* etiology	0.86 (0.11–6.92)	1.97 (1.28–3.04)	1.52 (1.12–2.07)	2.56 (2.03–3.22)

^a^No cases in Finland.

^b^The triad of death, severe neurological sequelae, or deafness.

In LatAm and Angola, underweight, late arrival, and pretreatment antibiotics also associated with higher mortality. With a weight/age z-score below -2, the odds ratio was 2.60 in LatAm *vs.* 1.48 in Africa. Having been ill for more than three days increased the odds ratio to 2.47 *vs.* 2.06, whereas pretreatment antibiotics increased the odds ratio to 1.21 *vs.* 1.42, respectively. Among the Finnish children, underweight, late arrival, or pretreatment antibiotics did not associate with death (wide 95%, CIs crossed 1). Controversially, age under one year increased fatality in LatAm, while it decreased it in Angola (OR 1.93 *vs.* 0.77). Anemia with hemoglobin less than 8.5 g/dl increased mortality in LatAm (OR 2.14).

Compared to death, the odds for dismal outcome were somewhat differentiated. While seizures increased the odds for dismal outcome in all sites (OR 6.87 in Finland, 3.33 in LatAm and 2.76 in Angola), in Finland a low Glasgow Coma score lost its significance, whereas weight/age z-score gained in significance. *S. pneumoniae* meningitis and anemia increased dismal outcome in LatAm and Angola, but not in Finland (OR in LatAm and Angola in pneumococcal meningitis 1.97 and 1.52; and when hemoglobin was < 8.5 g/dl 2.60 and 1.28).

Table 3 shows a multivariate regression analysis of prognostics factors for death and dismal outcome including study site as a categorical variable on the whole series. Study site, GCS < 13, seizures, and pneumococcal etiology were independent predictors of death. Study site, GCS < 13, seizures, age < 1 year, and pneumococcal etiology associated independently with dismal outcome.

**Table 3:** Odds ratios with 95% confidence intervals of variables related to death or dismal outcome, multivariate analysis.

Whole series	Death N = 1163		Dismal outcome^a^ N = 1058	
	OR (95% CI)	*p* Value	OR (95% CI)	*p* Value
LatAm vs. Finland	3.68 (1.59–8.53)	0.002	9.46 (4.35–20.61)	< 0.0001
Angola vs. LatAm	2.20 (1.42–3.42)	0.0004	1.26 (0.85–1.87)	0.26
Angola vs. Finland	8.12 (3.62–18.20)	< 0.0001	11.91 (5.54–25.63)	< 0.0001
Age < 1 year	-		1.55 (1.13–2.14)	0.007
Ill before arrival > 3 days	1.27 (0.90–1.78)	0.18	1.35 (0.97–1.89)	0.074
Pretreatment antibiotics	1.08 (0.78–1.51)	0.63	1.17 (0.85–1.62)	0.33
Weight/age z-score < -2	1.41 (0.98–2.02)	0.066	1.34 (0.93–1.93)	0.12
Seizures before/at arrival	1.95 (1.39–2.72)	0.0001	1.96 (1.43–2.69)	< 0.0001
Glasgow Coma Score < 13	3.41 (3.10–6.54)	< 0.0001	4.58 (3.31–6.32)	< 0.0001
Blood hemoglobin < 8.5 g/dl	1.18 (0.82–1.70)	0.36	1.29 (0.91–1.83)	0.15
*S. pneumoniae* etiology	1.40 (1.01–1.94)	0.044	1.49 (1.08–2.06)	0.015

^a^Death or severe neurological sequelae or deafness.

In Angola, the outcome of the small group of HIV positive children did not differ from the outcome of children with negative HIV test. Neither did the result of the malaria test affect the outcome.

## Discussion

The motive for our five trials that formed the study data was to improve prognosis of BM by modifying treatment. Since no real change in outcomes was observed, except for the notable finding of a significant reduction of severe neurological sequelae with glycerol in LatAm^12^, the accumulated dataset could be scrutinized as one, despite the treatments not being exactly the same. The long timespan may be seen as problematic, but a caveat is warranted: to our knowledge only one industrialized *vs.* non-industrialized country comparison on childhood BM has ever been published before^21^. So what conclusions can be drawn from our material?

First, no less than 11 of the 13 patient characteristics at arrival (Table 1) not only varied significantly between the sites, but most of them also associated with the outcomes. Altered consciousness was frequently observed across all series and demonstrated that children with BM generally arrived clinically very ill. More patients in Angola than LatAm scored under 13 on the Glasgow Scale, while the body temperature in Africa was lower. One may query whether widely used antibiotics in Luanda had already mitigated the disease, or if severe underweight in a third of the Angolan children had retarded adequate host response. For better or worse, dismal outcome was the sad fate for 54% of children in Angola, 31% in LatAm, and 5% in Finland—truly a somber finding. That seizures link to both death and dismal outcome is a lesser-known risk, whereas underweight^14^, delayed arrival^16^, and scoring low on the Glasgow Scale^13^ are among the recognized hazards for poor outcomes.

Second, three agents, Hib, *S. pneumoniae* and *N. meningitidis* held the same leading positions on all continents until vaccinations sharply reduced the role of Hib and pneumococci, first in Finland^11^ and a few years later in LatAm and Angola^22,23^. Since the African meningitis belt does not extend to Angola, an effective group A meningococcal conjugate^24^ has not been deployed there. Even fewer reasons for its use have been found in LatAm or Finland.

Third, pneumococcal meningitis was unsurprisingly more devastating than Hib or meningococcal meningitis (Fig. 3). However, our earlier analysis^13^ showed that more than etiology per se, it was the child's presenting condition that predicted the outcomes of BM. Since *S. pneumoniae* triggers a fierce inflammatory response^25^, pneumococcal meningitis often leads to terrible consequences. This would happen with any agent that caused an equally violent host response.

Fourth, tardy arrival to hospital worsened outcomes, especially in Angola, where median delay was five days, and a week not uncommon. This is almost incredible for such a life-threatening and often rapidly progressing disease as BM. Median delay in LatAm was three days, which is still longer than in Finland where patients usually arrived within 24 h or so. Interestingly, the pre-hospital duration of signs suggesting BM does not necessarily relate to outcome. In our group's earlier study on 325 patients from Finland^26^, no less than 26% of children had been sent home after a doctor's examination. Their outcomes did, however, not differ from the patients with swift diagnosis, no matter whether the delay had been 1 day, or 2 to 4 days.

Fifth, the negative effect of underweight, and to a slightly lesser extent of anemia, on dismal outcome were clear; obviously the lower the hemoglobin level, the poorer the outcome. Furthermore, AIDS, sickle cell disease, and parasitic infections are widely acknowledged problems, especially in Angola^27,28^. No wonder the risk of dying from BM in Malawi was five-fold compared to that in UK^21^.^.^ Here it was four-fold between LatAm (13%) and Finland (3.4%), and 11-fold between Angola (38%) and Finland. Such differences are unacceptable.

Sixth, performance of antibiotics was compared only in our first study in Finland^5^, but it is notable that new antibiotics have not improved the prognosis of BM, except in cases of resistance. Inexpensive ampicillin, and to a great extent even chloramphenicol—agents from the 1960s—successfully challenged third-generation cephalosporins^5^. Thus, local resistance pattern allowing, inexpensive β-lactams are still valid choices for at least pneumococcal and meningococcal meningitis. Costly antibiotics should be used only when truly needed. This is good news for resource-poor settings, where the incidence of BM remains highest.

Our analysis has expected limitations. The very prolonged timespan of data collection was a problem, but the rather stable nature of BM disease, and the prospective and uniform data recording was unlikely to have materially distorted the information obtained. Underlying diseases were potential confounding factors, especially in Angola. Ideally, all patients would have had full clinical and audiological investigation, up to perhaps one year post-hospitalization, as some problems may abate over time^29^. In the prevailing circumstances, however, late follow-up visits were feasible only in Finland. One may also argue that Finland does not represent all of Europe, nor Angola the whole of Africa. We deem those countries to be good representatives of their continents, however, despite the inter-country differences that certainly exist. The generalizability of the results was broadest for LatAm because several countries participated in the study. In general, the generalizability of our results corresponded to the time periods of the data collection, and may differ according to social and epidemiological circumstances. All patients did not always have 100% of the results from the analyzed variables. Therefore, exact numbers are presented in the Tables. Missing results were few and occurred at random and thus did not constitute a systemic selection bias.

Five large prospective studies using similar data collection protocols carried out in Scandinavia, Latin America and Africa generated a dataset of 2123 cases. Despite profound dissimilarities between the sites in their economies, resources and community life overall, as well as in availability of and access to health care, the causative agents were much the same. However, significant differences prevailed in some prognostically pivotal issues, such as days of illness before arrival to hospital, pre-treatment antibiotic use, history of seizures, altered consciousness, scoring on the Glasgow Coma Scale, severe anemia, and being underweight, all these being most frequent in Angola. Here, the children performed worst, as all these factors are associated significantly with adverse outcomes. Dismal outcome, comprising the triad of full deafness, severe neurological sequelae, or death, was the fate of 40% of children overall: for 54% of those in Angola, 31% in Latin America, and 5% in Finland. Poor outcomes in BM and their clear associations with local conditions persist as a major problem in resource-deficient settings. Fortunately, we are not without weapons, and so the fight must continue.

## Data Availability

The data that support the findings of this study are available from the corresponding author upon reasonable request.
